# Correction: Psychological Inoculation for Credibility Assessment, Sharing Intention, and Discernment of Misinformation: Systematic Review and Meta-Analysis

**DOI:** 10.2196/80134

**Published:** 2025-08-13

**Authors:** Chang Lu, Bo Hu, Qiang Li, Chao Bi, Xing-Da Ju

**Affiliations:** 1 School of Psychology, Northeast Normal University Changchun City, Jilin Province China; 2 Jilin Provincial Key Laboratory of Cognitive Neuroscience and Brain Development Changchun China

In “Psychological Inoculation for Credibility Assessment, Sharing Intention, and Discernment of Misinformation: Systematic Review and Meta-Analysis” (J Med Internet Res 2023;25: e49255) the authors made several clarifications to the Methods section to improve transparency.

The following textual amendments have been made to improve methodological clarity:

Under “Methods”, Eligibility Criteria (Textbox 1), item 6, the phrase:

Studies that included randomized controlled trials.

Has been replaced by:

Studies that included randomized controlled trials or quasi-experimental studies.

A new sentence has been added after this that contains a mention to a newly added Multimedia Appendix 4, which reads:

Removing Apuke 2022 [40], as the quasi-experimental study produced comparable pooled effects (see Multimedia Appendix 4).

Under “Methods”, Eligibility Criteria (Textbox 1), a new exclusion criterion has been added:

Studies did not include a control group.

Under “Methods”, Data Extraction, a definition paragraph has been added to the end of the section that reads:

Credibility discernment or sharing-discernment was included only when a study (a) reported a ready-made difference score between true and false items or (b) supplied the separate real information credibility assessment and misinformation credibility assessment (or real information sharing intention and misinformation sharing intention) statistics from which that difference could be calculated. If a study instead presented a single composite that mixed true and false items, regardless of reverse-scoring, that composite was coded as misinformation credibility assessment or misinformation sharing intention, not as a discernment outcome.

Under “Methods”, Data Analysis, first paragraph, 2 equations and 3 sentences have been inserted after the words, “For each study, means, SDs, and sample size were extracted. We computed a pooled effect size.” These equations and sentences read:

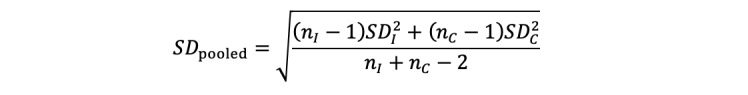

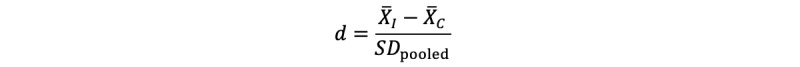

Where the subscripts *I* and *C* denote intervention and control groups. If the study did not report statistics, the Cohen *d* was calculated using *χ*^2^, *t*, η, and *F* values with the *Psychometrica* calculator and the *Campbell* calculator. When a study did not provide the group-level mean, standard deviation, or sample size required for calculating Cohen d, we contacted the corresponding author to request those statistics. When authors supplied only an overall sample size, we divided that total equally between the two arms. Four studies were queried, but none of the authors responded. In such cases we reconstructed effect sizes from the information that was available.

The correction will appear in the online version of the paper on the JMIR Publications website, together with the publication of this correction notice on August 13, 2025. Because this was made after submission to PubMed, PubMed Central, and other full-text repositories, the corrected article has also been resubmitted to those repositories.

## Appendix

Multimedia Appendix 1 PRISMA (Preferred Reporting Items for Systematic Reviews and Meta-Analysis) checklist.

Multimedia Appendix 2 Search strategies.

Multimedia Appendix 3 Characteristics of Studies Included in the Meta-analysis.

Multimedia Appendix 4 Supplementary analysis of excluded Apuke 2022.

Multimedia Appendix 5 Code of Supplementary analysis.
