# Supplementary material for: Correction: Psychological Inoculation for Credibility Assessment, Sharing Intention, and Discernment of Misinformation: Systematic Review and Meta-Analysis
Source: J Med Internet Res. 2025 Aug 13;27:e80134. doi: 10.2196/80134 (PMC12391837; doi:10.2196/80134)

Forest plot for the effects of psychological inoculation on misinformation information credibility (exclude Apuke 2022)


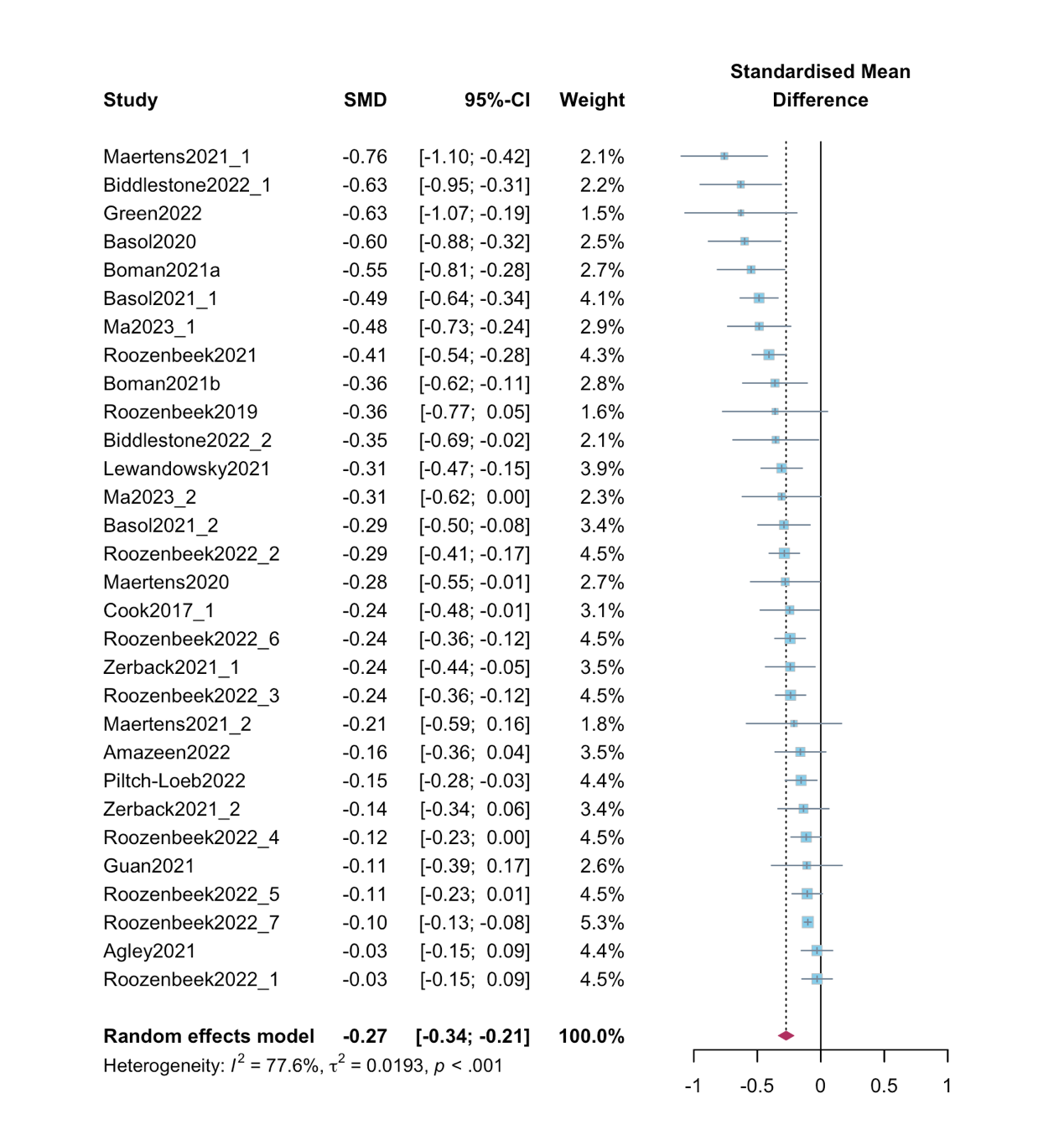


Forest plot for the effects of psychological inoculation on misinformation information sharing intention (exclude Apuke 2022)


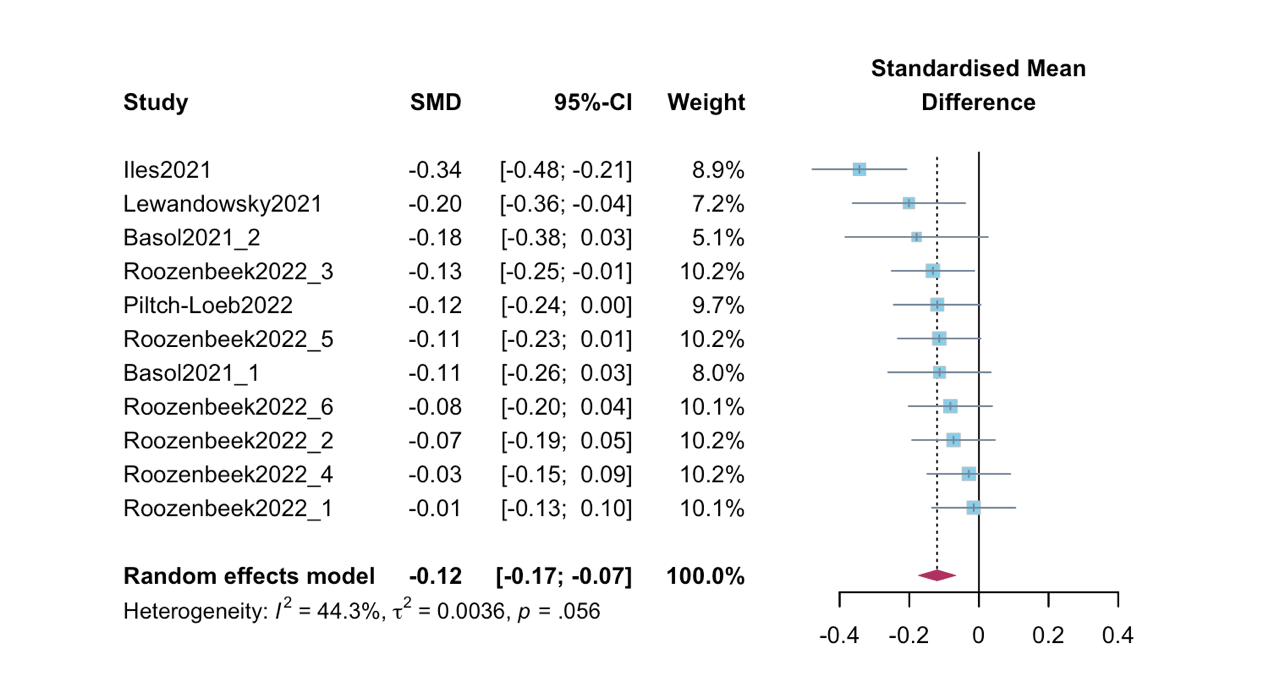

Supplement: Multimedia Appendix 4 [file jmir_v27i1e80134_app4.docx]
