# Supplementary figures and images for: Correction: Psychological Inoculation for Credibility Assessment, Sharing Intention, and Discernment of Misinformation: Systematic Review and Meta-Analysis
Source: J Med Internet Res. 2025 Aug 13;27:e80134. doi: 10.2196/80134 (PMC12391837; doi:10.2196/80134)

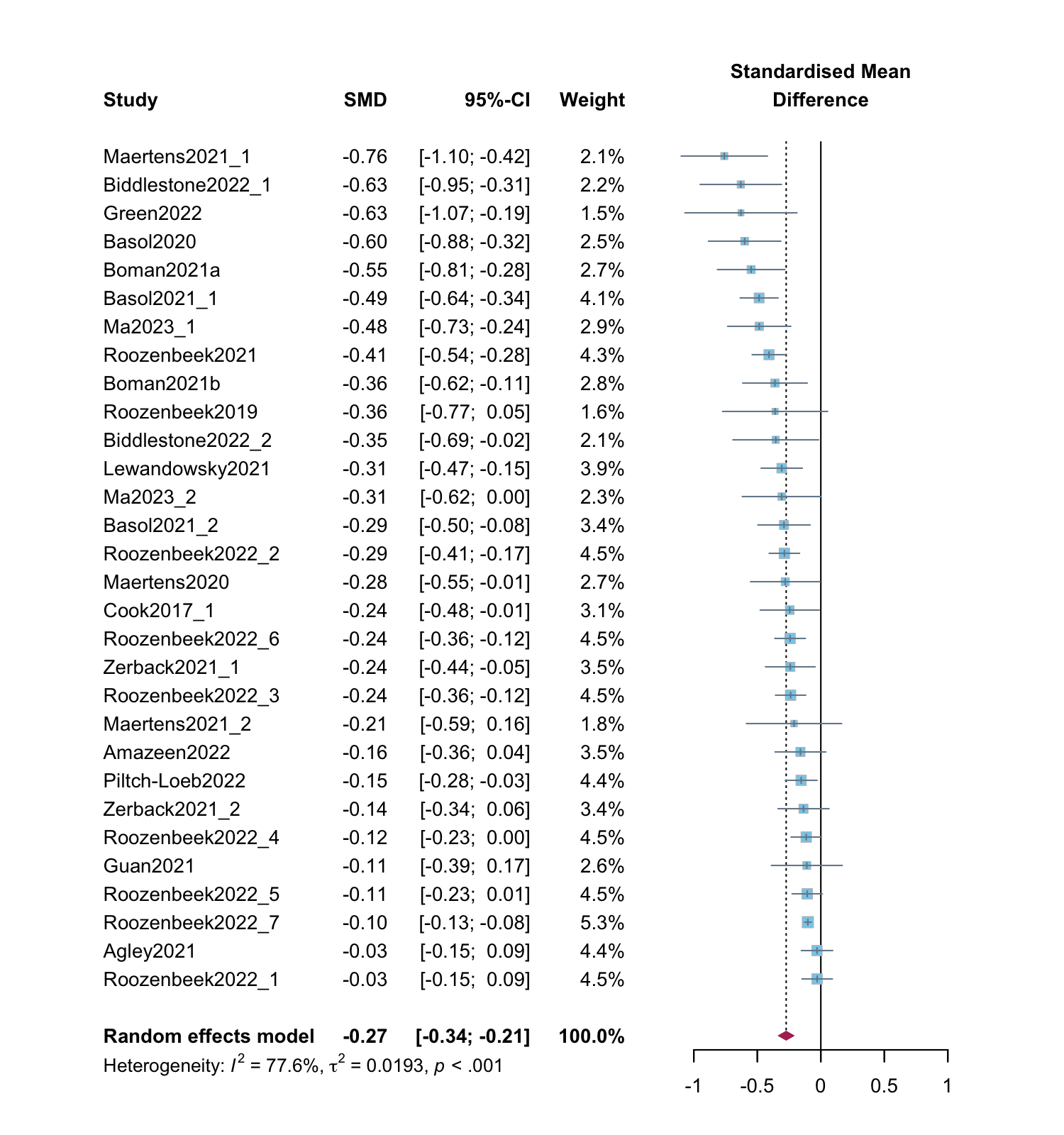

Supplement: Multimedia Appendix 5 [file jmir_v27i1e80134_app5.zip › Supplementary analysis/Misinformation_Credibility/forest.tiff]

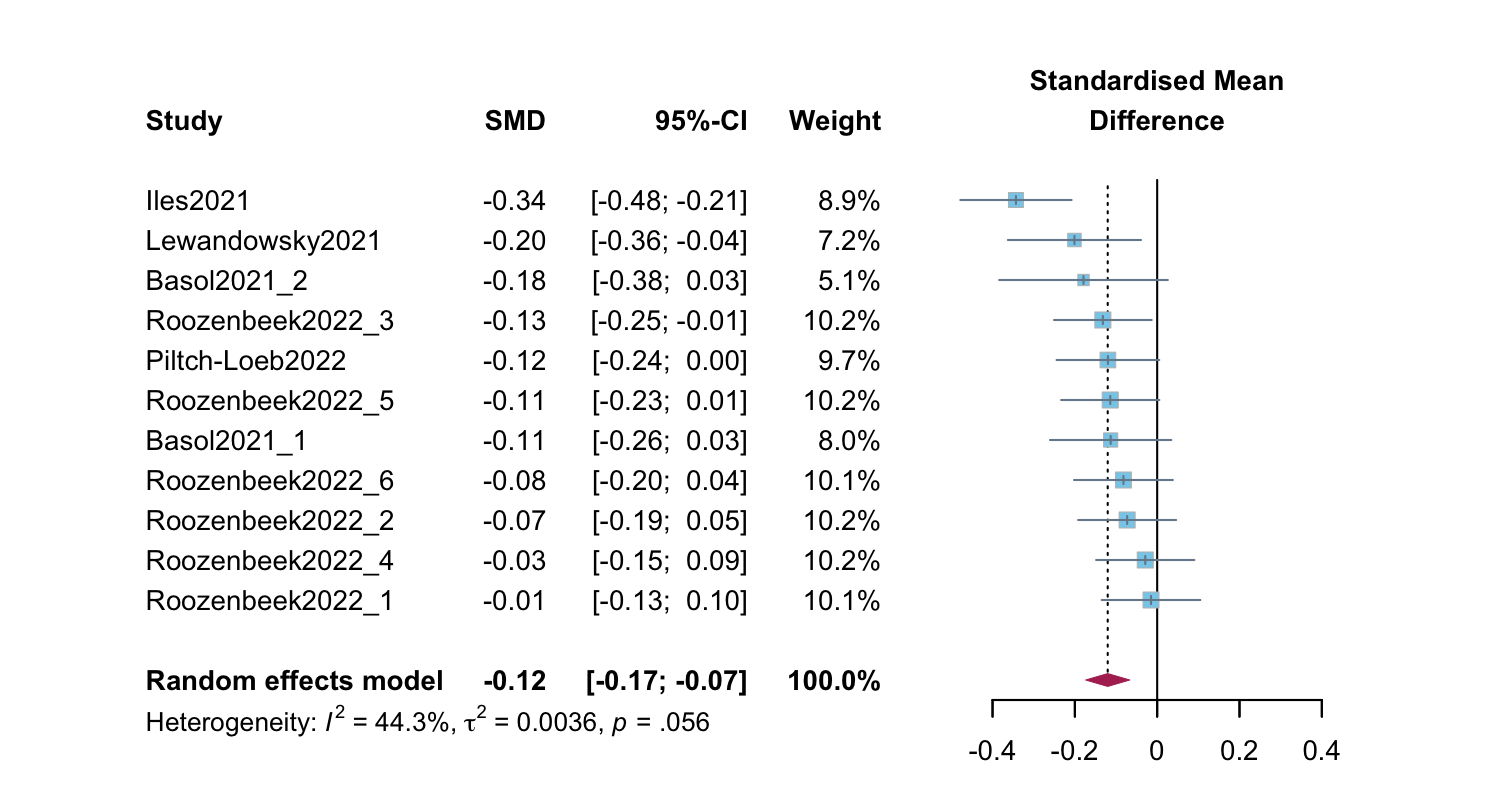

Supplement: Multimedia Appendix 5 [file jmir_v27i1e80134_app5.zip › Supplementary analysis/Misinformation_Sharing/forest.tiff]
